# Design and evaluation of a data anonymization pipeline to promote Open Science on COVID-19

**DOI:** 10.1038/s41597-020-00773-y

**Published:** 2020-12-10

**Authors:** Carolin E. M. Jakob, Florian Kohlmayer, Thierry Meurers, Jörg Janne Vehreschild, Fabian Prasser

**Affiliations:** 1 University Hospital Cologne, Cologne, Germany; 2 School of Medicine, Technical University of Munich, Munich, Germany; 3 Berlin Institute of Health (BIH), Berlin, Germany; 4 Charité – Universitätsmedizin Berlin, corporate member of Freie Universität Berlin, Humboldt-Universität zu Berlin, and Berlin Institute of Health, Berlin, Germany; 5 German Center for Infection Research (DZIF), partner site Bonn-Cologne, Cologne, Germany; 6 Department of Internal Medicine, Hematology and Oncology, Goethe University Frankfurt, Frankfurt am Main, Germany

**Keywords:** Data publication and archiving, Software, Epidemiology

## Abstract

The Lean European Open Survey on SARS-CoV-2 Infected Patients (LEOSS) is a European registry for studying the epidemiology and clinical course of COVID-19. To support evidence-generation at the rapid pace required in a pandemic, LEOSS follows an Open Science approach, making data available to the public in real-time. To protect patient privacy, quantitative anonymization procedures are used to protect the continuously published data stream consisting of 16 variables on the course and therapy of COVID-19 from singling out, inference and linkage attacks. We investigated the bias introduced by this process and found that it has very little impact on the quality of output data. Current laws do not specify requirements for the application of formal anonymization methods, there is a lack of guidelines with clear recommendations and few real-world applications of quantitative anonymization procedures have been described in the literature. We therefore believe that our work can help others with developing urgently needed anonymization pipelines for their projects.

## Introduction

### Background

The novel coronavirus, Severe Acute Respiratory Syndrome Coronavirus II (SARS-CoV-2), and its associated respiratory illness COVID-19 can spread from person to person^1^. SARS-CoV-2 was declared a public health emergency of international concern by the end of January and a pandemic by mid of March 2020. The WHO continuously publishes knowledge about the outbreak and stresses the necessity for in-depth research^2^. Until SARS-CoV-2 and COVID-19 have been sufficiently well understood and effective medication or even a vaccine has been developed, non-pharmaceutical interventions, such as social distancing, travel restrictions, closing of public institutions and businesses, quarantines and curfews are alternative options^3,4^. Many of these measures have drastic socio-economic consequences^5^. For example, it has been estimated that in the Euro area the impact of the reduction in economic activity as a result of lockdowns will be up to 3–4 larger than that of the global financial crisis of 2007/2008^6^. Hence, there is a significant need for rapid access to research data on the novel coronavirus.

The Lean European Open Survey on SARS-CoV-2 Infected Patients (LEOSS)^7^ has been established in March 2020 as a European non-interventional multi-center cohort study with the aim to overcome the lack of knowledge about epidemiology and clinical course of SARS-COV-2, to further develop evidence-based diagnostic and therapeutic recommendations and to determine independent outcome predictors. The registry collects data on hospitalized patients and patients who receive ambulant medical consultation of all ages. As of July 2020, more than 125 sites from 7 different countries have been registered to LEOSS and more than 2.500 patients have been included. Daily statistics are provided on the LEOSS website (https://leoss.net).

To provide data at the rapid pace required in a pandemic, LEOSS implements a special concept. It consists of autonomous, self-managed study sites that collect data in an anonymous form. To achieve this, no directly-identifying data is stored in the registry and demographic data as well as timestamps are only collected in a coarsened form. Approval for LEOSS was obtained by the local ethics committees of all participating centers and the study was registered at the German Clinical Trails Register (DRKS, No. S00021145). To ensure data quality, the registry requires registration, authentication and authorization for data entry. Data collection is only performed once per case, retrospectively after treatment has finished or the patient has died. While this obviously impedes the ability to collect longitudinal data and to follow-up discharged patients, it does have the benefit that no informed consent is necessary. Furthermore, this enables the inclusion of data about children and unconscious or deceased patients and avoids problems that could arise from language barriers. Moreover, non-university hospitals and private practices often do not have the resources to obtain and archive informed consent forms.

The LEOSS registry follows an Open Science approach to make data available to the community in real-time^8^. This is in contrast to other multi-centric COVID-19 registries, such as the WHO ISARIC 4 C consortium (https://isaric4c.net/), that solely regulate data access through applications to data access committees (DACs). A notable example of another project publishing open clinical data on COVID-19 is the 4CE consortium^9^. However, the data published by this project is aggregated on a country level. LEOSS makes selected data items available in real-time without aggregation and without the need of an application process.

Within LEOSS a dual strategy is pursued to implement an Open Science approach as well as traditional means of data access. First, a Public Use File (PUF) is provided as an open dataset on the project website that can be freely accessed by researchers and citizen scientists alike. Second, more comprehensive Scientific Use Files (SUFs) are provided to the scientific community upon request to a DAC under the condition that researchers sign a Data Use Agreement (DUA) and properly protected the dataset. In both cases, anonymization procedures are applied to the datasets to protect the data from re-identification and further risks of privacy breaches. The PUF currently contains 16 variables, while the SUFs can contain up to 605 variables. As the dimensionality of data has a significant impact on the effectiveness of anonymization techniques different approaches are applied to both datasets^10^.

### Objectives

The objective of the work described in this article was to develop a quantitative anonymization pipeline for the LEOSS PUF. Relevant requirements are laid out in national and international laws and regulations, including, for example, the U.S. Health Insurance Portability and Accountability Act (HIPAA)^11^ and the European General Data Protection Regulation (GDPR)^12^. However, no specific recommendations for the application of formal and statistical methods is provided and there is a lack of guidelines with clear recommendations regarding the specific design of quantitative anonymization processes^13^. The need to protect health data sufficiently, often represents a significant barrier that is difficult to overcome in data sharing endeavors. Analogously, there are only very few publications describing the development process and design of formal data anonymization pipelines for real-world data sharing projects (an overview is provided in the Section “Discussion”). As a result, we had to overcome several challenges while developing the pipeline for the PUF:

1. Anonymization involves the modification of data, which affects its statistical properties and thus the accuracy of the conclusions drawn on its basis^14^. We therefore had to carefully calibrate the methods used for measuring risks as well as the transformations applied, to ensure that the PUF remains useful and at the same time complies with GDPR-specific requirements for publishing an open dataset.
2. While the LEOSS registry is maintained at the University Hospital Cologne, the anonymization pipeline has been developed in cooperation with partners in Berlin and Munich. Hence, we had to use a development methodology that allowed external data protection experts to design the anonymization process without access to the primary dataset.
3. The LEOSS PUF is updated regularly. Hence, we needed to continuously monitor our data publishing activities to ensure that the method originally developed continued to work well for updated datasets.

## Results

### Public use file

The LEOSS PUF is generated from applying the anonymization pipeline on the primary data of LEOSS. An overview of the dataset, including basic patient demographics, phases of COVID-19 the patient has gone through, therapy, intervention, incidence of superinfections, and symptoms, is provided in Online-only Table 1.

### Anonymization pipeline

In addition to the LEOSS PUF, we believe that the design of the anonymization pipeline as well as its development process are the most important contributions of our work. For this purpose, we will summarize these aspects and refer to the Section “Methods” for further details.

We utilized a process consisting of three basic steps which was able to involve external partners into the implementation of the anonymization pipeline: (1) a synthetic file generated from the LEOSS dataset was used to develop an initial version of the pipeline, (2) the pipeline was executed on the dataset to generate a report containing information on the effect of anonymization procedures on the dataset, (3) this report was used to adjust the pipeline with the aim to improve the quality of the anonymized output dataset. Steps 2 and 3 were executed seven times over the course of six weeks (March to April 2020), until the final version of the anonymization pipeline had been developed.

To elicit the anonymization requirements to be fulfilled by the pipeline, qualitative and quantitative risk assessment methods were utilized. First, a qualitative assessment of the 16 variables selected for publication was performed. For this purpose, attributes form the LEOSS PUF were compared to lists of risky variables provided in anonymization standards and guidelines, including the Safe Harbor method of the Privacy Rule of the US HIPAA law^15^ (18 specific variables) and the European Medicines Agency (EMA)^16^ Policy 007 Implementation Guideline for anonymous sharing of clinical trials data (two types of variables, i.e. dates and locations). As the dataset only contained one risky variable mentioned in these guidelines (“Month first diagnosis”), it was concluded that the privacy risk of publishing the LEOSS PUF would be low, even in its original form.

Next, a qualitative risk assessment was performed to derive requirements for formal guarantees on the degree of protection needed and to follow recommendations that have been made by the Article 29 Data Protection Working Party (the predecessor of the European Data Protection Board) regarding anonymization under the GDPR^17^. Following the recommendations of the Working Party, we derived requirements to protect the data from (1) singling out, i.e. the possibility to isolate records belonging to an individual, (2) linkability, i.e. the possibility to link records belonging to one or multiple individuals, and (3) inference, i.e. the possibility to deduce sensitive information about individuals.

A privacy model suggested by the Working Party for mitigating the risk of singling out and linkage is k-anonymity^17^, which requires that each record is indistinguishable from at least k-1 other records regarding attributes that could be used to directly or indirectly identify individuals^13^. To determine these attributes, a methodology proposed by Malin *et al*. was used in which the replicability, availability and distinguishability of data is analyzed^18^. Four attributes exceeded the predefined risk threshold (“Age at diagnosis”, “Gender”, “Month first diagnosis”, “Year first diagnosis”) and hence needed to be protected (more details are provided in the Section “Quantitative risk assessment and anonymization procedures”). As a value for the privacy parameter, k = 11 was chosen, which follows recommendations by the Working Party^17^ and the EMA guideline^16^.

The Working Party suggested additional mitigating controls to be implemented for risks of inference. It was decided to require that all remaining attributes are protected from inference. For this purpose, the t-closeness privacy model was used^19^, which has also been recommended by the Working Party^17^. This model requires that the distribution of sensitive data for individual records or groups of records does not deviate too much from the overall distribution. As a parameter t = 0.5 was selected, which takes into account the high level of privacy protection already achieved. In addition, it was required that all values contained in the PUF must correspond to at least 10 individuals. This requirement was already been described in the LEOSS study protocol^8^ and provides an additional layer of protection for individuals with rare characteristics.

The PUF is continuously updated as new primary data is entered into the registry. This needs to be considered when implementing anonymization processes^14^. For example, simply anonymizing all newly added records and appending them to the PUF can lead to datasets that do not meet the protection requirements^19^. Hence, it was required that the anonymization process must be robust in this regard. This was achieved by always executing the pipeline on the complete primary file and holding back all records that do not fulfill the protection requirements defined. The pipeline was implemented using the open source ARX Data Anonymization Tool^20^.

### Evaluation

**Fig. 1:**
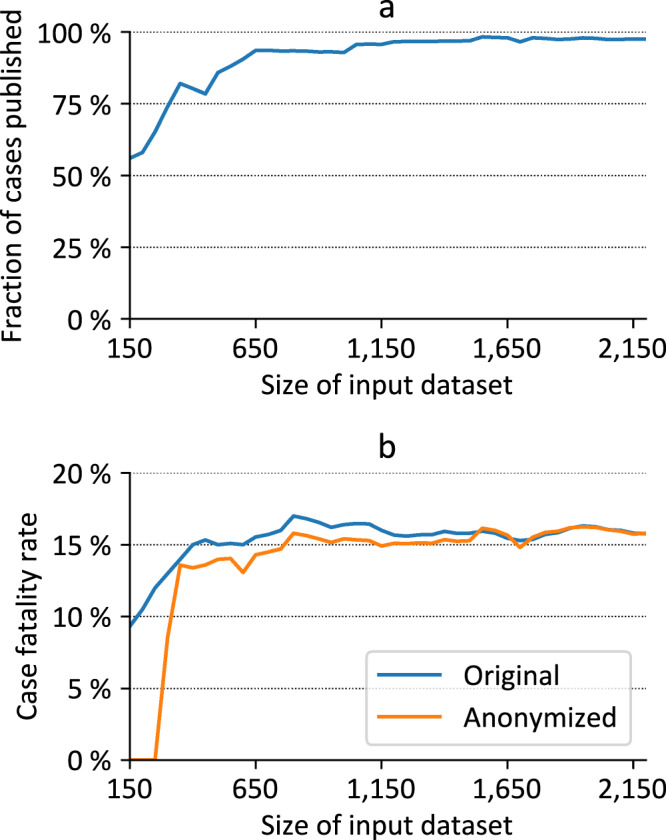
Development of various properties of the LEOSS PUF over time, which is represented by the size of the primary dataset in LEOSS. (**a**) Fraction of cases published, (**b**) case fatality rate before and after anonymization.

The privacy models implemented by the pipeline are based on the principle of “hiding in the crowd”. This means that the privacy of patients is protected by making sure that their information does not differ significantly from the information about a larger group of individuals. For this reason, the anonymization pipeline developed has an interesting and somewhat counter-intuitive property: the greater the number of individuals included in the LEOSS registry, the less information has to be removed from the LEOSS PUF to achieve the required degree of protection^21,22^. We considered this fact when designing the pipeline and accepted a higher degree of removed records and hence bias in initial release. Fig. 1 presents an overview of how selected properties of the PUF have changed over time.

As seen in Fig. 1(a), the number of cases that needed to be removed from the primary dataset has decreased over time. In the beginning (03/30/2020), when only 150 cases were documented in the registry, over 40% of the records could not be published. Currently, as of 06/09/2020, only 2.5% of the 2,200 cases documented in LEOSS need to be excluded from the PUF.

Fig. 1(b) provides an example of the statistical bias caused by the removal of records in the released file. The initial LEOSS PUF did not contain any cases in which patients had died from COVID-19. However, the Case Fatality Rate (CFR) calculated using the primary data from the registry and the CRF calculated using the PUF quickly converged. Already with 500 cases documented in the registry, the difference was only 1.01%. Using 2,200 cases in the primary dataset, the CFR calculated from the PUF is only overestimated by 0.03%.

**Fig. 2:**
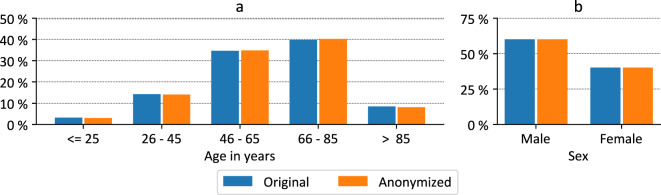
Comparison of demographic parameters before and after anonymization for the primary dataset with 2,200 records. (**a**) Age distribution in years, (**b**) gender distribution.

**Fig. 3:**
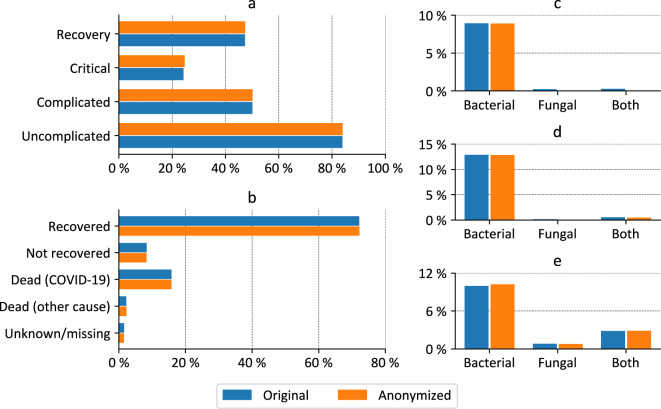
Comparison of clinical parameters before and after anonymization for the primary dataset with 2,200 records. (**a**) Patients per phase, (**b**) outcome, (**c**) superinfections in uncomplicated phase, (**d**) superinfection in complicated phase, (**e**) superinfections in critical phase.

Figs. 2 and 3 provide more detailed comparisons between the primary LEOSS dataset, including 2,200 records and the PUF (2,145 records). Fig. 2(a) shows a comparison of the age distributions. There are only minor differences which are caused by the suppression of particularly young (aged <  = 25 years) or old (aged > 85 years) patients. The gender distributions shown in Fig. 2(b), are equal with 59.95% men and 40.05% women.

Fig. 3(a–e) show comparisons of selected clinical parameters. As can be seen, there are only minor differences (on average, frequencies differ by only 0.11%).

Differences between the PUF and the primary data stem from the fact that some patients with very specific characteristics (patients with fungal or bacterial and fungal superinfections) have been excluded from the PUF. However, in total, this affects only 14 out of the 2,200 (0.64%) cases documented. Further differences can be found in the distribution of phases amongst the cases with the most significant difference being that the number of cases going through a critical phase increased from 24.27% in the primary dataset to 24.71% in the PUF.

**Fig. 4:**
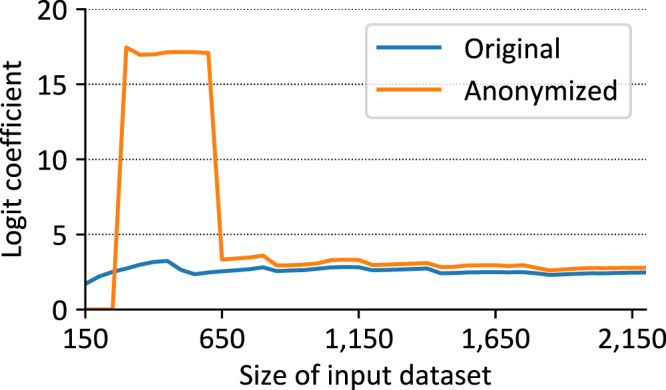
Change of logit coefficients of univariate association of patients with age >45 years and death before and after anonymization over time, which is represented by the size of the primary dataset.

We also studied the impact of anonymization on further statistical analyses. An example is provided in Fig. 4, which shows the development of logit coefficients for the univariate regression model between age and death before and after anonymization over time. The primary dataset started to show a significant association with 200 records (200 records: coefficient: 2.51; p-value: 0.033; 2,200 records: coefficient: 2.46; p-value: <0.001). However, the PUF showed a significant association only with more than 650 records (650 records: coefficient: 3.33; p-value: <0.001; 2,200 records: coefficient: 2.78; p-value: <0.001). Reasons for the high logit coefficients between the 300 and 600 datasets are that just in the primary dataset of 650 records the first patient aged < 45 years who died was included. The patient also remained in the PUF. The association of age and death is overestimated in the PUF. This can be explained, as patients aged < 45 years are less frequently included in LEOSS (Fig. 2(a)). Therefore, also the probability being excluded by the anonymization pipeline is higher.

**Fig. 5:**
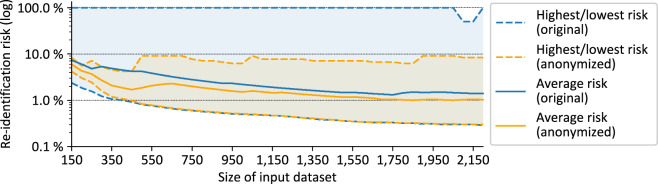
Development of re-identification risks before and after anonymization.

Fig. 5 provides insights into the effectiveness of the anonymization pipeline in managing the privacy risks of the records in the PUF. The figure shows the lowest, highest and average re-identification risk of the records in the processed primary datasets and the anonymized output datasets produced, which is calculated from the size of the groups of indistinguishable records in which they are included. It can be seen that the primary datasets always included records with a very high re-identification risk, while the output dataset never contained any records with a risk higher than 9.09% (which reflects the requirement of 11-anonymity). At the same time, it can be seen that the lowest re-identification risk of any record basically remained the same in both datasets and that also the average re-identification risk was only reduced slightly (by up to 2.77%) while the difference decreased over time. This means that the anonymization pipeline has served exactly the purpose of protecting those records that were at too high a risk, while minimizing the impact on the remainder of the dataset.

## Discussion

In this article, we have presented a robust and rigorously designed anonymization pipeline for releasing an open dataset from a research registry on COVID-19. Moreover, we have presented a practical process for involving data protection experts into the development of anonymization pipelines for medical research projects. Our application of quantitative methods was based on qualitative analyses of the data to be published, best practices and recommendations from laws and guidelines. By combining protection against singling out and linkage with additional protection against inference of sensitive information, the resulting anonymized dataset is strongly protected from the threats addressed by relevant laws and regulations. Moreover, the initially strong impact of anonymization on the quality of the dataset has declined quickly, and the current version of the PUF is very close to the primary dataset. The LEOSS PUF is made available on the project website with regular updates since 04/12/2020.

Established anonymization guidelines only provide general information on available methods and pitfalls to consider^15,16,23^. Moreover, prior scientific articles that describe anonymization procedures applied to real-world datasets typically focus on qualitative measures only. One example is a paper describing the anonymization process applied to data from a trial investigating the use of mercaptopurine in chronic disease^24^. The process was based on guidelines published by the UK Medical Research Council (MRC) Hubs for Trials Methodology Research (HTMR)^25^ and involved PIs, statisticians and programmers that decided by consensus which variables should be deleted or modified. In contrast to our approach, no formal protection guarantees can be derived from this process.

Regarding quantitative methods, the NHS has provided an open dataset on potential symptoms of COVID-19 that have been reported through emergency calls^26^. Based on the experiences gathered while creating this file, the Open Data Institute has provided a guideline on data anonymization^27^. However, neither the guideline nor the NHS provided specific details and recommendations regarding privacy models and risk thresholds. In another study, Kuzilek *et al*. have described the anonymization process applied to create the Open University Learning Analytics Dataset, which is a representative subset of student data collected at the Open University. The data was also anonymized using ARX in a process that has been certified by the Open Data Institute^28^. Compared to the work described in this article, the dataset contains significantly more records and attributes. To create the public dataset, a random sample has been taken, which would not be acceptable in our context. Moreover, only k-anonymity with k = 5 has been utilized to protect a subset of the attributes from susceptibility to linkage attacks. No protection against sensitive attribute disclosure has been implemented. However, in contrast to the work described in this article, this is seen as acceptable as the data is much less sensitive than the medical data managed by the LEOSS registry.

We agree with several experts in the field that “there is a need for anonymization standards that can provide operational guidance to data custodians and promote consistency in the applications of anonymisation”^13^. This has become even more important in times of pandemic. For example, the Global Research Collaboration for Infectious Disease Preparedness (GloPID-R) has developed a roadmap in which it is stated that “anonymisation of data is of high importance and appropriate protocols need to be put in place […]“^29^. We strongly believe that our description of the development methodology utilized and pipeline implemented for the LEOSS registry can be a vital resource helping other researchers with developing urgently needed data sharing procedures for their projects.

## Methods

### Development method

The LEOSS registry is maintained by clinical experts at the University Hospital Cologne (LEOSS team). Data is entered via a web-based user frontend to an Electronic Data Capture (EDC) system.

**Fig. 6:**
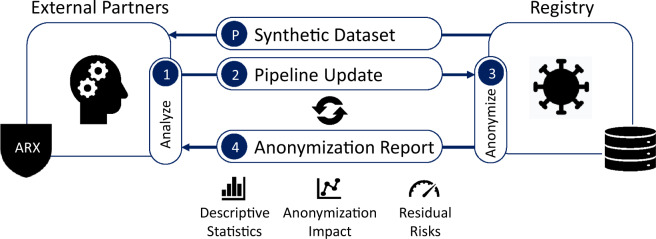
Overview of the workflow used to develop the anonymization pipeline.

The anonymization pipeline has been developed in cooperation with external partners at Charité Berlin, the Berlin Institute of Health and the University Hospital rechts der Isar of the Technical University of Munich, which could not be granted access to the primary data collected in LEOSS. While developing the pipeline, we therefore repeatedly followed the process outlined in Fig. 6.

In a preparatory step, the LEOSS team agreed on the set of variables that should be contained in the PUF. The LEOSS team then created a synthetic dataset out of the according subset of primary data available in the LEOSS registry. For this purpose, a set of 1,000 records was generated by randomly drawing values from the domains of the variables in the primary dataset. The resulting dataset captured the basic characteristics of the PUF (variables, domains) but did not reflect the original data distribution and hence did not pose any privacy risks (non-personal data). This synthetic dataset was then provided in encrypted form via Email to the external partners working on the anonymization pipeline. From this point on, we repeatedly executed the following steps:

- **Step 1:** The external partners analyzed the synthetic dataset and/or a report and updated the data anonymization pipeline. The pipeline was designed in such a way that it outputs an anonymized dataset as well as a report describing the impact of the different transformation procedures implemented on re-identification risks and the quality of output data.
- **Step 2:** The (updated) pipeline was provided to the LEOSS team via a shared software repository (Git^30^) hosted at the University Hospital of Cologne.
- **Step 3:** The LEOSS team executed the pipeline on the primary dataset, checked the anonymized output dataset and collected the report generated.
- **Step 4:** Information on errors that appeared during the anonymization process, problems with the anonymized output dataset as well as the report generated by the pipeline was sent via Email to the teams in Berlin and Munich. Important information contained in the report included descriptive statistics of the variables in the dataset before and after applying different anonymization steps as well as information on records associated with a high risk of re-identification as well as further estimates of residual risks. At this point, the process was repeated with step 1.

In the following sections, we describe the qualitative and quantitative risk assessment procedures utilized in this process, the requirements derived from the results of these assessments as well as details on our implementation.

### Anonymization method

#### Qualitative risk assessment

As a first step, we performed a qualitative risk assessment of the variables selected for publication, an overview of which is provided in Online-only Table 1. From a qualitative perspective we concluded that the dataset contains no directly identifying information and only a very small number of variables that are typically assumed to be associated with a high risk of re-identification (“Age at diagnosis”, “Gender”, “Month first diagnosis”, “Year first diagnosis”). Notably, the dataset only contains one variable, “month first diagnosis”, that would need to be removed according to the de-identification standard laid out in the Safe Harbor method of the Privacy Rule of the US HIPAA law^15^ (note that “Age at diagnosis” is top-coded at 85) or that is considered a high-risk variable by the European Medicines Agency’s Policy 007 Implementation Guideline for anonymous sharing of clinical trials data^16^. There are multiple studies indicating that the risk of re-identification of HIPAA protected data is very small^31^. From a qualitative perspective, we therefore concluded that the privacy risk of publishing the LEOSS PUF is low, even in its original form.

#### Quantitative risk assessment and anonymization procedures

Next, we defined additional requirements for qualitative risk assessment and anonymization procedures to be able to provide formal guarantees on the degree of provided protection and to follow recommendations that have been made regarding anonymization under the GDPR.

When implementing quantitative risk management techniques, we followed the requirements described by the Article 29 Data Protection Working Party, which was an advisory body composed of a representative of the data protection authority of each EU Member State, the European Data Protection Supervisor and the European Commission that became the European Data Protection Board with the introduction of the GDPR. With its “Opinion on Anonymisation Methods”^17^ the board formulated requirements and guidelines for effective anonymization measures and presented an assessment of common methods. According to the opinion, the following privacy threats need to be addressed by anonymization methods^17^:

- Singling out: “the possibility to isolate some or all records which identify an individual in the dataset“^17^
- Linkability: “the ability to link, at least, two records concerning the same data subject or a group of data subjects“^17^
- Inference: “the possibility to deduce, with significant probability, the value of an attribute from the values of a set of other attributes”^17^

To assess which variables must be transformed to protect records from singling out and linkability, we implemented the approach proposed by Malin et al. and analyzed the replicability, availability and distinguishability (quantified by 1 = low, 2 = medium, 3 = high) of the variables^18^. Thereafter, the result of this analysis was used to estimate how well suited these variables would be for successfully performing linkage attacks (if sum of weights is >5; we called those “key” variables). The results are shown in Table 1.

**Table 1:** Assessment of the re-identification risk associated with individual variables.

Variable	Replicable	Available	Distinguish.	Key
Age at diagnosis	3	3	3	Yes (9)
Gender	3	3	2	Yes (8)
Month first diagnosis	1	3	2	Yes (6)
Year first diagnosis	1	3	2	Yes (6)
Uncomplicated phase	1	2	1	No (4)
Complicated phase	1	2	2	No (5)
Critical phase	1	2	2	No (5)
Recovery phase	1	2	1	No (4)
Vasopressors in complicated phase	1	1	2	No (4)
Vasopressors in critical phase	1	1	2	No (4)
Invasive ventilation in critical phase	1	1	2	No (4)
Superinfection in uncomplicated phase	1	1	2	No (4)
Superinfection in complicated phase	1	1	2	No (4)
Superinfection in critical phase	1	1	2	No (4)
Symptoms in recovery phase	1	1	2	No (4)
Last known patient status	1	2	2	No (5)

As can be seen in Table 1, our analysis indicated that basic demographics and timestamps are information that is likely known to potential adversaries and could be used in linkage attacks. This is consistent with guidelines and recommendations^15,25^. Hence, we needed to prevent singling out and linkability using the variables “Age at diagnosis”, “Gender”, “Month first diagnosis”, “Year first diagnosis” or any arbitrary combination. A privacy model suggested by the opinion for mitigating this risk is k-anonymity^17^. This well-known model requires that each record is indistinguishable from at least k-1 other records regarding the key variables, i.e. variables that could be used for dataset linkage^21^. In terms of parametrization, the Working Party recommends a value of k > 10, which is consistent with recommendations from other guidelines, including the European Medicines Agency’s Policy 007 Implementation Guideline^16^, that recommends a risk threshold of 9.09% (corresponding to k = 11). We therefore derived the following requirement:

**Requirement 1:** The LEOSS PUF must satisfy k-anonymity with k = 11 regarding the variables “Age at diagnosis”, “Gender”, “Month first diagnosis” and “Year first diagnosis”.

While the opinion states that the risk of inference is also partially addressed by 11-anonymity, it still recommends additional protection. Since the data collected in LEOSS is sensitive medical information, we decided to protect all remaining (i.e. non-key) variables from inference. To implement this, we decided to utilize the t-closeness privacy model^19^, which has also been recommended by the opinion^17^. The model requires that the distribution of sensitive attribute values within a cluster of indistinguishable individuals is not too different from the distribution of those values in the overall dataset. As is described in more detail in the “Results” section, this is enforced by transforming the key variables. Some variables, in particular those describing whether patients went through a particular phase, are perfectly correlated with the variables describing complications, interventions and symptoms (i.e. their value can be derived from the fact whether information on complications, interventions or symptoms has been provided for the according phase). As protection for the more detailed medical variables is implemented by transforming the key variables, these variables will automatically be protected as well. Regarding the parametrization we chose t = 0.5, which takes into account the high level of privacy protection already achieved.

**Requirement 2:** The LEOSS PUF must satisfy t-closeness with t = 0.5 for the sensitive variables “Vasopressors in complicated phase”, “Vasopressors in critical phase”, “Invasive ventilation in critical phase”, “Superinfection in uncomplicated phase”, “Superinfection in complicated phase”, “Superinfection in critical phase”, “Symptoms in recovery phase” and “Last known patient status”.

In addition, we decided to require that all values contained in the PUF must correspond to at least 10 individuals, which is a requirement that has already been described in the LEOSS study protocol^8^. While this provides little formal guarantees, it does provide an additional layer of protection for individuals with rare characteristics regarding individual variables:

**Requirement 3:** The LEOSS PUF must not contain any attribute values that correspond to less than 10 individuals.

The PUF is updated continuously while new primary data is entered into the registry. To ensure that all data remains adequately protected, this needs to be considered when implementing anonymization processes^14^. For example, simply anonymizing all newly added records and appending them to the PUF can lead to datasets that do not meet the protection requirements described in this section^19^. Hence we defined the following final requirement:

**Requirement 4:** The anonymization pipeline for the LEOSS PUF must guarantee that the protection requirements 1–3 are fulfilled, even when data is published continuously.

### Implementation

On the conceptual level, privacy requirements can be enforced using a wide variety of methods, including data generalization, aggregation or randomization^14^. For creating the LEOSS PUF we decided to use a simple approach, in which the pipeline just removes records from the primary dataset that do not fulfil the privacy requirements defined. While higher quality output data could have potentially been achieved by using more sophisticated methods, this approach also has two important advantages. First, it is non-pertubative, which means that the removal of records is the only potential source of bias thus facilitating statistical analyses. Second, when this process is always applied to the complete LEOSS primary dataset (not just newly added records) it also fulfils the requirement that the protection requirements are met even when data is published continuously.

**Fig. 7:**
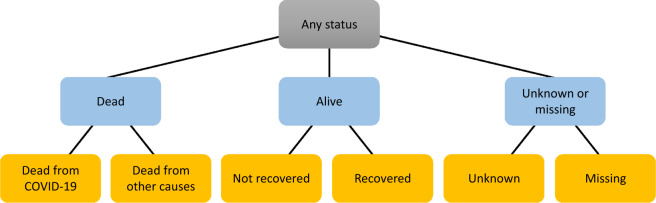
Semantic domain structuring for a sensitive variable.

Another design decision we had to make was how exactly to measure risks. In particular, t-closeness is a privacy model for which several variants have been proposed that use different methods for measuring the distance between distributions^19^. To measure this aspect as accurately as possible and thus obtain the highest possible output data quality, we chose a variant that considers the semantic relationship between values^19^. For this purpose, we developed hierarchical structurings of the domains of all sensitive variables. An example for the variable “Last known patient status” is shown in Fig. 7.

On the technical level, we used the open source ARX Data Anonymization Tool to implement the pipeline^20^. The software is mature and robust, has been mentioned in a variety of official reports and guidelines^16,32^ and used to create open datasets, such as the Open University Learning Analytics Dataset (OULAD)^28^.

### Evaluation method

As mentioned before, anonymization procedures can have significant impacts on the quality and statistical properties of data. To ensure that the LEOSS PUF remains to meet high quality standards, we used the reports generated by the anonymization pipeline to closely monitor the data publishing activities. The generated reports contained the distributions of attribute values in input and output data as well as numbers on the impact of transformations performed by the pipeline on records and values in the dataset. In this paper, we used those numbers as well as comparisons of the results of inferential statistical methods to provide insights into the quality of the LEOSS PUF and how it has evolved over time.

### Limitations and future work

The LEOSS PUF has been designed as an open dataset that can be made freely accessible to the public. To protect privacy in this context, it comprises a carefully selected set of variables providing a general overview of the course of COVID-19 and it is protected by strong anonymization methods. As a consequence, its utility for answering more complex scientific questions is limited.

As mentioned above, LEOSS also makes more comprehensive and detailed SUFs available to researchers upon request. As these datasets can contain up to 605 variables, a different anonymization process must be implemented. First SUFs have already been created and shared with the scientific community. In this process we built upon the development method and tools described in this article, but used less formal anonymization methods and followed “rules of thumb” to protect the data as much as possible while ensuring that the datasets fit the requesting researchers’ requirements. This process is time-consuming, as relevant requirements vary from request to request. Moreover, additional protection measures, such as DUAs, need to be implemented. In future work, we plan to further streamline this process by implementing different anonymization modules for different parts of the data managed by LEOSS. We hope that these modules can then be flexibly combined to generate SUFs for different researchers more efficiently. If this approach works well, we plan to summarize the design of these modules in an anonymization guideline describing best practices for generating SUFs for medical datasets.

## Data Availability

The PUF is published as a CSV file on the data section of the LEOSS website (https://leoss.net/data/). Moreover, an interactive dashboard is provided that displays a net graph visualizing the incidence and course of clinical phases of COVID-19 patients based on the LEOSS PUF (https://dashboard.leoss.net/). The current version at the time of writing has been archived online^33^.

## Online-only Table

**Online-only Table 1:** Overview of the variables in the LEOSS PUF.

Variable	Description	Domain
Age at diagnosis	Age of patient at time of diagnosis	<=25 26 - 45 46 - 65 66 - 85 > 85
Gender	Sex of patient	Male Female
Month first diagnosis	Month of first confirmed diagnosis of COVID-19	1–12
Year first diagnosis	Year of first confirmed diagnosis of COVID-19	4 digit year
Uncomplicated phase	Indicates whether the patient has been through the uncomplicated phase of COVID-19	Yes No
Complicated phase	Indicates whether the patient has been through the complicated phase of COVID-19	Yes No
Critical phase	Indicates whether the patient has been through the critical phase of COVID-19	Yes No
Recovery phase	Indicates whether the patient has been through the recovery phase of COVID-19	Yes No
Vasopressors in complicated phase	Indicates whether vasopressors were used in the complicated phase	Yes No Missing/unknown N/a
Vasopressors in critical phase	Indicates whether vasopressors were used in the critical phase	Yes No Missing/unknown N/a
Invasive ventilation in critical phase	Indicates whether invasive ventilation was used in the critical phase	Yes No Missing/unknown N/a
Superinfection in uncomplicated phase	Type of (if any) superinfection in uncomplicated phase	Bacterial Fungal Bacterial & fungal None Missing/unknown N/a
Superinfection in complicated phase	Type of (if any) superinfection in complicated phase	Bacterial Fungal Bacterial & fungal None Missing/unknown N/a
Superinfection in critical phase	Type of (if any) superinfection in critical phase	Bacterial Fungal Bacterial & fungal None Missing/unknown N/a
Symptoms in recovery phase	Symptoms (if any) in recovery phase	Yes No Missing/unknown N/a
Last known patient status	Last known patient status	Recovered Not recovered Dead from COVID-19 Dead from other causes Unknown/missing

n/a; not applicable. For definitions of COVID-19 phases see leoss.net/statistics

## Code availability

The source code of the anonymization pipeline developed for the LEOSS PUF is publicly available as open source software^34^.
